# Ergothioneine, collagen peptides, and sodium hyaluronate ameliorate facial skin aging via modulating oxidative stress, inflammation, and extracellular matrix homeostasis

**DOI:** 10.3389/fnut.2026.1888269

**Published:** 2026-08-21

**Authors:** Zhiqing Zhou, Lingling Zhao, Lei Chen, Xuanxiang Zhao, Xinwei Li, Xuying Luo, Bin Zhang, Jianyin Miao, Feitong Liu

**Affiliations:** 1 Overseas Expertise Introduction Center for Discipline Innovation of Food Nutrition and Human Health, School of Food Science and Engineering, South China University of Technology, Guangzhou, China; 2 China Research and Innovation Center, H&H Research, H&H Group, Guangzhou, China; 3 Guangdong Provincial Key Laboratory of Nutraceuticals and Functional Foods, College of Food Science, South China Agricultural University, Guangzhou, China; 4 School of Food Science and Engineering, South China University of Technology, Guangzhou, China

**Keywords:** anti-aging, collagen peptides, ergothioneine, extracellular matrix homeostasis, facial skin aging, oral nutricosmetic, oxidative stress, sodium hyaluronate

## Abstract

**Background:**

Skin aging involves intrinsic chronological aging and extrinsic photoaging, characterized by impaired barrier function, extracellular matrix (ECM) degradation, and chronic inflammation.

**Methods:**

This study evaluated anti-aging efficacy and mechanisms of ergothioneine (EGT), collagen peptides (CP) and sodium hyaluronate (NaHA) using an *ex vivo* human skin model and a clinical trial.

**Results:**

*Ex vivo* results showed that the EGT+CP+NaHA triple combination (TC) attenuated UV-induced epithelial thinning and collagen fiber disruption. Mechanistically, TC enhanced antioxidant capacity by up-regulating Nrf2 and SOD2 expression, modulated the senescence-associated secretory phenotype (SASP) via restoring TGF-β1 and suppressing IL-1α, IL-6, and MMP1 levels, and preserved ECM integrity by reversing reductions in collagen I, III, IV, VII, XVII, and hyaluronic acid (HA) contents. Clinically, oral TC to Chinese women improved skin hydration, elasticity, and barrier function, while reducing the number, depth, area, and volume of crow's feet wrinkles, nasolabial folds and marionette lines.

**Conclusion:**

These findings demonstrate that TC exerts multifaceted anti-aging effects by targeting oxidative stress, inflammatory pathways, and ECM homeostasis, providing a scientific basis for the development of novel oral nutricosmetics for facial skin anti-aging.

## 1 Introduction

Skin aging represents the most prominent and visually impactful hallmark of aging, profoundly influencing individuals' aesthetic perception and quality of life. It is orchestrated by two intertwined and mutually reinforcing processes, intrinsic chronological aging and extrinsic photoaging, that collectively drive characteristic structural, functional, and molecular alterations in the skin (1). Intrinsic aging, a naturally progressive process, is marked by gradual epidermal thinning, diminished sebaceous and sweat gland activity leading to skin dryness, reduced elastic fiber resilience, and the formation of fine, superficial wrinkles (1–3). In contrast, extrinsic photoaging, primarily triggered by cumulative ultraviolet (UV) radiation exposure, is characterized by more severe and distinct manifestations such as irregular pigmentation, skin roughness, deep wrinkles, elastosis, and impaired barrier function (4, 5). A common underpinning of both aging pathways is the overproduction of reactive oxygen species (ROS), which induces oxidative stress, DNA damage, and a state of chronic low-grade inflammation, ultimately disrupting the homeostasis of the dermal ECM (1, 6). Given the multifactorial and interconnected nature of skin aging, single-ingredient interventions often fail to address its complex mechanisms comprehensively, highlighting the urgent need for superior oral combination strategies that target multiple pathogenic pathways simultaneously.

EGT, a naturally occurring hydrophilic antioxidant and unique thiol derivative, has emerged as a promising candidate in anti-aging research (7). Beyond its free radical scavenging ability, studies have linked EGT to supporting brain function (8–10), alleviating depressive symptoms (11, 12), and protecting against central nervous system diseases (13–15). As a nutricosmetic ingredient, EGT has been incorporated into various topical skincare products and oral supplements due to its stability and safety profile (16). Preliminary *in vitro* studies have demonstrated that EGT can mitigate UV-induced fibroblast senescence and oxidative damage (17, 18). However, conclusive *in vivo* evidence confirming its efficacy in improving clinically relevant facial skin aging phenotypes, particularly when combined with other bioactive ingredients, remains scarce.

CP, especially low-molecular-weight fractions (< 3,000 Da) with high bioavailability and gastrointestinal absorption efficiency, has been extensively validated for their anti-aging effects on facial skin (19–21). The most recognized mechanism involves the promotion of collagen and elastin synthesis in the skin, coupled with a reduction in collagen fragmentation through the inhibition of matrix metalloproteinases (MMPs) (22, 23). Low-molecular-weight CP also enhances dermal density, skin hydration, and elasticity, thereby reducing the appearance of wrinkles, melanin formation, and inflammation, and improving skin integrity and counteracting aging (21, 24–27). Despite their widespread use as oral nutricosmetics, optimizing their efficacy through combinations with complementary bioactive ingredients remains a key research gap.

HA, commonly utilized in the form of NaHA for its superior water solubility and biocompatibility, is a major component of the skin ECM that plays a pivotal role in maintaining tissue hydration, elasticity, and structural integrity (28, 29). Current clinical studies on NaHA predominantly focuses on its topical and injection applications (30–32). Dietary supplementation with NaHA is primarily recognized for improving skin hydration (28), with recent studies reporting NaHA's efficacy in alleviating aging-related dry skin and wrinkles in Western populations (29). However, research on the anti-aging effects of NaHA on facial skin, particularly in Asian populations, remains lacking. Furthermore, the potential effects of NaHA in combination with CP and EGT in modulating skin aging pathways have not been systematically explored.

To address these research gaps, the present study aimed to investigate the anti-aging efficacy and underlying mechanisms of a novel oral combination of EGT, CP and NaHA. A comprehensive dual-approach strategy was employed: first, an *ex vivo* human skin model was used to evaluate the protective effects against UV-induced photoaging, focusing on epidermal thickness, collagen fibers, the expression of key collagens, and biomarkers related to skin hydration and antioxidant capacity. Second, the clinical anti-aging benefits were tested in a healthy population of Chinese women aged 32–54, focusing on improvements in dermal collagen density, skin elasticity, smoothness, and the visibility of crow's-feet wrinkles, nasolabial folds, and marionette lines. Through investigations into both UV-induced photoaging and natural chronological aging, this research provides systematic evidence for the dermatological benefits of the TC.

## 2 Method

### 2.1 *Ex vivo* human skin model building

Abdominal skin samples from 32- or 36-year-old female donor (GT-0003015 and GT-0003528, BIOCYTO, China) were immersed in 75% alcohol for 30 s followed by washing with PBS and then cut into 0.6 cm diameter circular tissue pieces as the model. This study and the use of human skin samples were approved by the Ethics Committee (GDLL2025003 and GDLL2025006). The models were cultured in 6-well plates with 3.7 mL medium in a 37 °C incubator with 5% CO_2_. After 2 days of culture, models were assigned to eight groups. All groups except the non-irradiated Control received fresh medium supplemented with specific ingredients and were then exposed to 30 J/cm^2^ UVA and 50 mJ/cm^2^ UVB (PL-S 9W/10/2P and PL-S 9W/01/2P, Philips, Netherlands). The irradiated groups were: Model (medium only), vitamin C (VC; 0.01%; w/v; A7506, Sigma, USA) + vitamin E (VE; 0.0007%; w/v; 238813, Sigma, USA), CP (JELLICE, Singapore; 0.08%; w/v) in which over 90% of components have an average molecular weight below 10,000 Da, and more than 20% are collagen tripeptide, low-molecular-weight NaHA (about 320 kDa; BLOOMAGE BIOTECH, China; 0.005%; w/v), EGT (Shanghai EGT Synbio Group Co., Ltd., China; 0.002%; w/v), EGT + CP, EGT + NaHA, and EGT + CP + NaHA at the same concentrations. During culture, the epidermal part of the models is exposed to air while the rest is immersed in the culture medium. The intervention was performed by adding ingredients into the medium. This UV treatment was repeated daily for four consecutive days, followed by 3 days of culture without irradiation.

### 2.2 H&E staining and Masson staining

After the treatment as mentioned above, the models were fixed with 4% paraformaldehyde for 24 h and then subjected to hematoxylin and eosin (H&E) staining (C0107 and C0105S, Biyotime, China) or Masson's trichrome staining (YK2223, COUAL BIO, China). The images were captured using an upright microscope system (BX53, Olympus, Japan).

### 2.3 Immunofluorescence

The skin explants were fixed with 4% paraformaldehyde for 24 h, embedded, sectioned, and then blocked for 1 h at room temperature. The tissue sections were incubated with primary antibodies, including type I collagen (72026S, CST, USA), type III collagen (22734-1-AP, Proteintech, China), type IV collagen (ab6311, Abcam, UK), type VII collagen (ab309143, Abcam, UK), type XVII collagen (ab186415, Abcam, UK), AQP3 (ab125219, Abcam, UK), TGF-β1 (ab170874, Abcam, UK), Hyaluronan Binding Protein (Biotin Conj.) for detecting HA (AMS.HKD-BC41, Amsbio, UK), SOD2 (ab13534, Abcam, UK) and Nrf2 (ab62352, Abcam, UK), at 4 °C overnight. Following primary antibody incubation, the sections were incubated with appropriate secondary antibodies (ab150117 and ab150077, Abcam, UK) and imaged by a fluorescence microscope system (CKX53, Olympus, Japan).

### 2.4 ELISA

The concentration of IL-1α, IL-6 and MMP1 was detected using commercial ELISA kits, IL-1α ELISA kit (ab100560, Abcam, UK), IL-6 ELISA kit (ab46027, Abcam, UK) and MMP-1 ELISA kit (ab215083, Abcam, UK) according to the manufacturers' instructions. Absorbance was measured at the appropriate wavelength with a microplate spectrophotometer (Epoch, BioTek, USA).

### 2.5 Clinical evaluation of facial skin appearance

A mono-centric clinical study was conducted in Qingdao, China from August to October 2025. The study protocol was approved by the local ethics committee (ethics approval QDMED-WI-011-R03) and strictly adhered to the principles of the Declaration of Helsinki. All subjects provided written informed consent prior to participation. Trial registration: ClinicalTrials.gov, ChiCTR2600123703 (retrospectively registered on 29 Apr. 2026).

**Figure 1:**
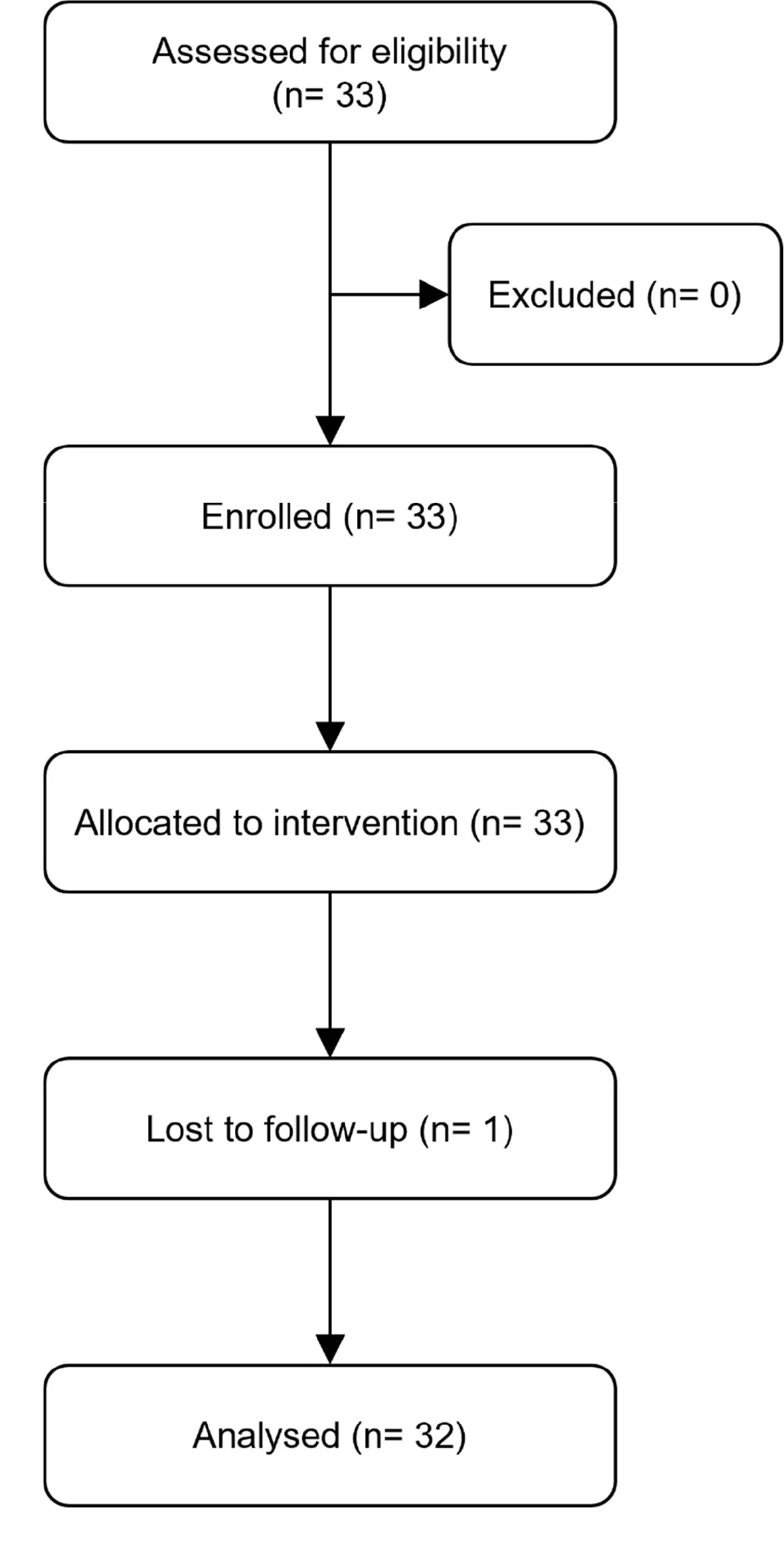
Study flowchart.

Thirty-two Chinese female volunteers, aged 30–55 with all skin types, were recruited. This clinical trial began in August 2025 and ended in October 2025. All subjects presented with facial redness but had no obvious skin lesions, scars, or facial hair. They were required to have a habit of daily sun protection and maintain their previous skincare routines during this clinical trial. Specific data regarding patient recruitment, allocation and follow-up are presented in Figure 1.

Skin qualities were adjusted based on SGS Asian Atlas (15). Inclusion criteria were as follows: cheek skin hydration (< 60 C.U.); cheek skin transepidermal water loss rate (>15 g/h/m^2^); crow's feet wrinkles (3 ≤ grade ≤ 6, in Atlas); nasolabial folds (3 ≤ grade ≤ 6, in Atlas); marionette lines (3 ≤ grade ≤ 6, in Atlas); facial skin glossiness (3 ≤ grade ≤ 6, in Atlas). Exclusion criteria included: any active skin disease or allergy (particularly infectious diseases or atopic dermatitis) in the test areas; ongoing use of medications that might interfere with the study outcomes.

Subjects were asked to take three tablets orally once a day for 8 weeks. This regimen corresponded to a daily intake of 25 mg EGT, 2,500 mg CP, 120 mg NaHA. Assessments were performed at 0 week (D0), 1 week (D7), 2 weeks (D14), 4 weeks (D28), and 8 weeks (D56). Prior to those assessments, subjects acclimatized to ambient conditions for 30 min. Standardized facial photographs (frontal, left, and right profiles) under all lighting modes were captured using VISIA-7 (Canfield, USA). Skin's elasticity and firmness were measured by Cutometer (Courage & Khazaka, Germany). Skin hydration was measured by Corneometer (Courage & Khazaka, Germany), and transepidermal water loss (TEWL) was measured by Tewameter (Courage & Khazaka, Germany). Skin roughness was measured by Visoscan^®^ VC20plus (Courage & Khazaka, Germany). Skin thickness and ultrasound density were measured by DermaLab^®^ Combo (Cortex Technology, Denmark).

### 2.6 Statistical analysis

Statistical analyses and mapping were performed using Statistics 28.0 (IBM Corporation, Chicago, IL, USA). Data from *in vitro* experiment were expressed as mean ± SD. The control group and model group were analyzed using a *t*-test. For the other groups, Levene's test was used to assess the homogeneity of variances, and the Shapiro–Wilk normality test was used to confirm normal distribution. Inter-group differences were evaluated using one-way ANOVA, followed by Dunnett's test for comparing the other groups with the control group. For the clinical trial data, results are presented as mean ± SD. Normality was assessed using the Shapiro-Wilk test. Based on the normality test results, paired *t*-tests or Wilcoxon signed-rank tests were applied for within-group comparisons across time points. Significant level α = 0.05.

## 3 Result

### 3.1 EGT, CP, and NaHA reduce the skin damage induced by UV exposure

**Figure 2:**
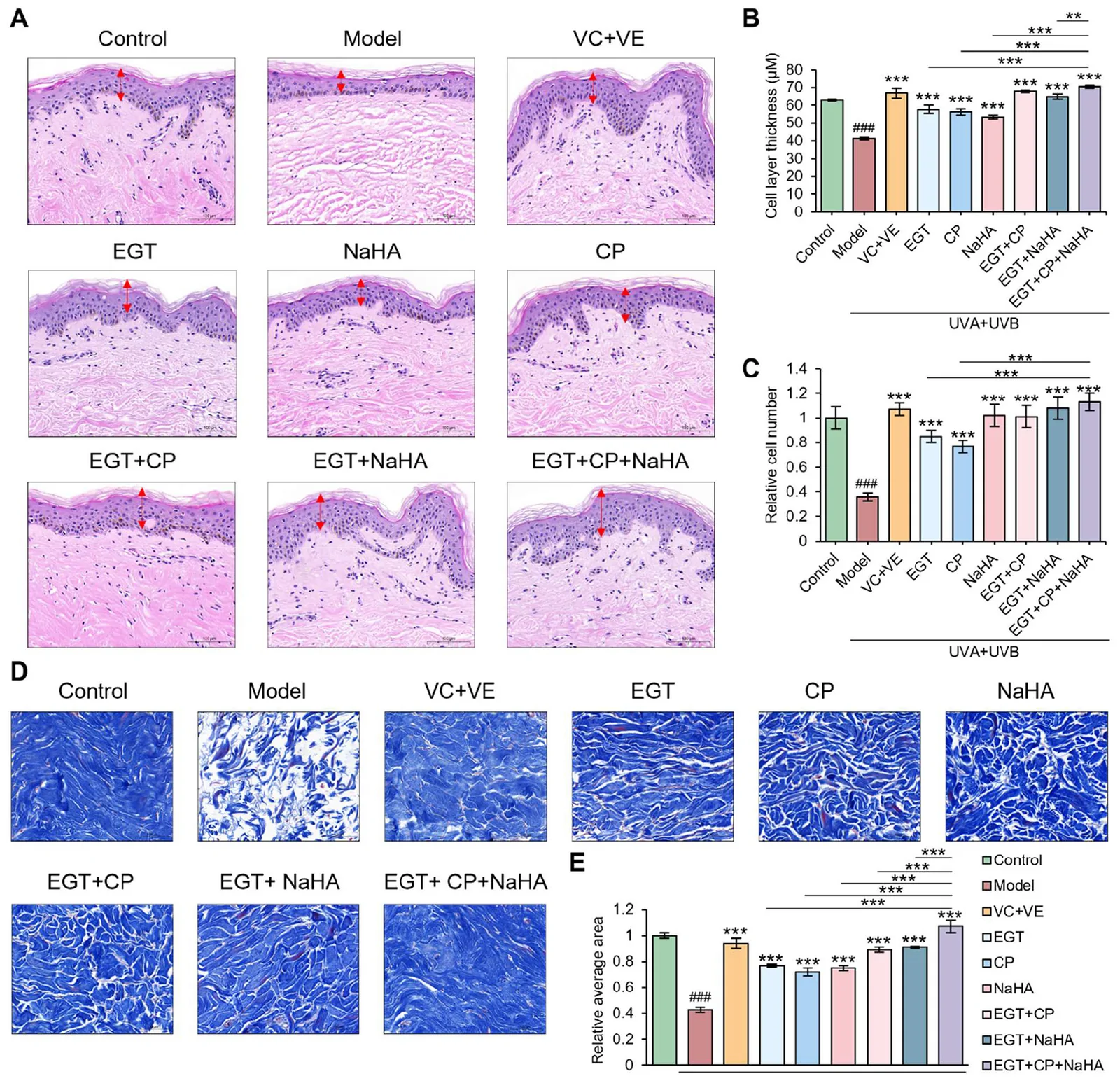
EGT, CP, and NaHA protected against UV-induced skin barrier damage and mitigated the reduction of collagen fiber content in 3D human epidermis model. Except for the control group, all groups were treated with UVA and UVB irradiation and *n* = 3. **(A)** Morphology of tissues revealed by H&E staining, the red bidirectional arrow mark indicates the cell layer thickness; **(B)** thickness of the living cell layers; **(C)** relative cell number (cell number per unit area of living cell layers); **(D)** collagen fibers visualized by Masson's staining; **(E)** relative average area of collagen fiber. Statistical analysis was performed using Student's *t*-test. ### Indicates *p*-value <0.001 compares with control groups; ** indicates *p*-value <0.01, *** indicates *p*-value <0.001 compares with model groups.

The success of the UV-induced photoaging model was verified by hematoxylin and eosin (H&E) staining, which showed a significant thinning of the viable epidermal layer in the UV-irradiated Model group compared to the non-irradiated Control group (Figures 2A–C). Notably, the positive control (VC+VE) group exhibited a marked reversal of this UV-induced damage, confirming the model's validity and the feasibility of intervention (Figures 2A–C).

All the tested ingredients, whether applied alone (EGT, CP, NaHA) or in combination, significantly preserved cell layer thickness and cell number per unit area of cell layer from UV damage (Figures 2A–C). The combination of EGT, CP and NaHA showed enhanced efficacy over any single ingredient and dual combinations (Figure 2B).

Consistent with the H&E findings, Masson's staining showed that the UV-induced loosening and breakage of collagen fibers were reversed by those compounds, with the combinations—particularly the triple combination TC—proving most effective (Figures 2D, E). Those results suggest that EGT, CP, and NaHA protect the skin barrier and the integrity of collagen fibers against damage induced by UV exposure.

Collectively, these findings indicate that EGT, CP, and NaHA each exerts protective effects against UV-induced skin barrier damage and collagen fiber degradation. Importantly, the triple combination demonstrates a higher protective effect that surpasses individual or dual-component treatments, highlighting its potential as a multifaceted intervention for UV-induced photoaging.

### 3.2 EGT, CP, and NaHA reverse UV-induced reduction of ECM

The ECM is a crucial component of skin, filling the intercellular space and serving as a physical scaffold that maintains tissue integrity (33–35). To further confirm the protective effect of TC against UV-induced skin photoaging, we conducted immunofluorescence assay to quantify the levels of key ECM molecules in an *ex vivo* human skin model.

**Figure 3:**
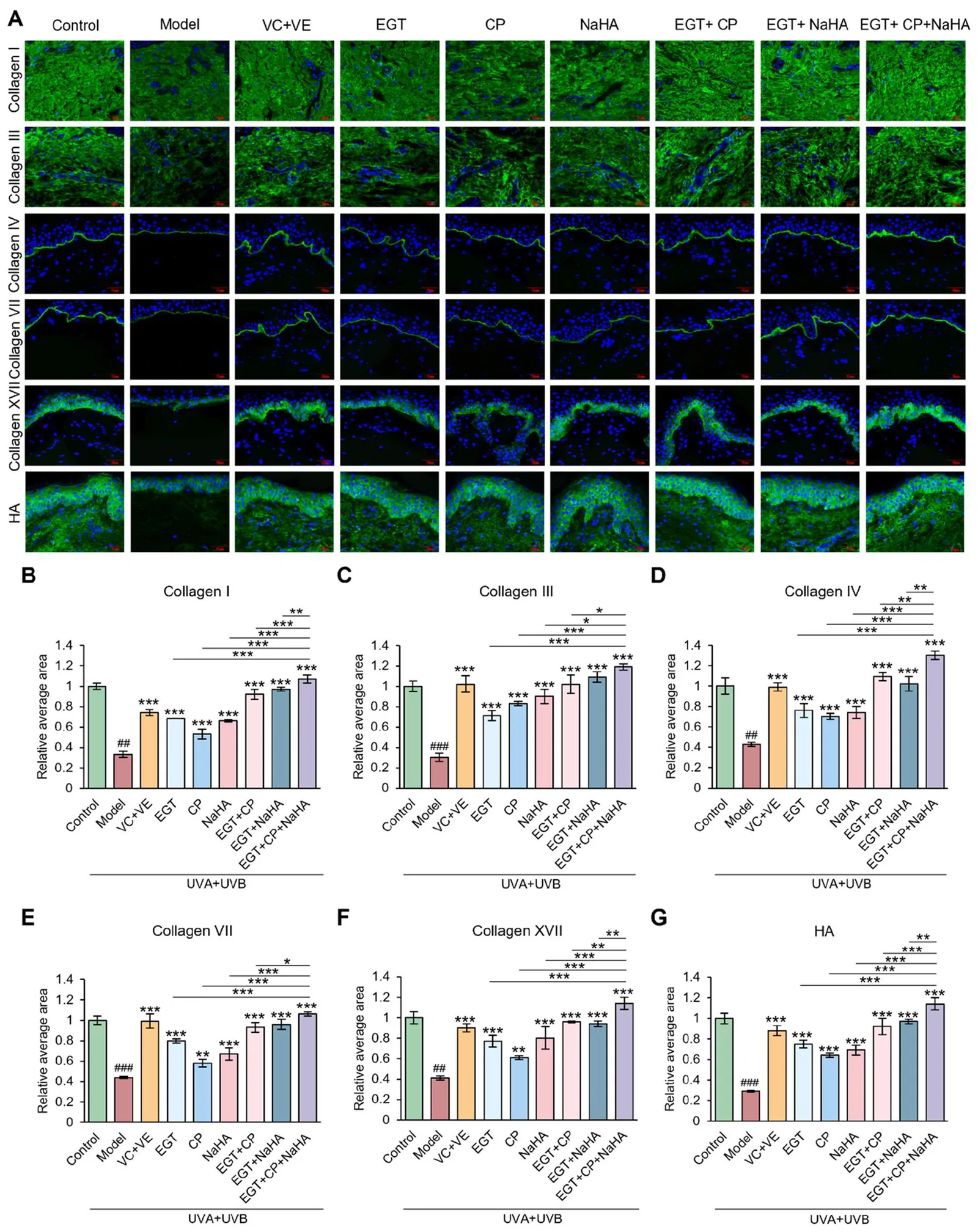
The concentration of ECM in skin model with UV irradiation. **(A)** Immunofluorescence results show the concentration of ECM components, including collagen I, collagen III, collagen IV, collagen VII, collagen XVII, and HA with fluorescence intensity; the relative concentration of **(B)** collagen I, **(C)** collagen III, **(D)** collagen IV, **(E)** collagen VII, **(F)** collagen XVII, and **(G)** HA. Samples in every groups *n* = 3. Statistical analysis was performed using Student's *t*-test. ## Indicates *p*-value <0.01, ### indicates *p*-value <0.001 compares with control groups; * indicates *p*-value <0.05, ** indicates *p*-value <0.01, *** indicates *p*-value <0.001 compares with model groups.

Collagen is the main ECM building blocks (33), with type I collagen accounting for 80%−90% and type III collagen for 8%−12% of the total collagen content in the skin (36). These two collagens are essential for maintaining skin structural integrity, elasticity, and tensile strength (34). Our experiments demonstrated that UV irradiation significantly reduced collagen I and collagen III levels to 67% and 70% of the control values, respectively, and the TC treatment restored these levels to 107% and 119% of baseline with the TC treatment (Figures 3A–C), indicating robust protection of skin structural integrity and physiological function.

UV irradiation also damages the dense ECM layer known as the basement membrane (BM) (37), which anchors the epidermis and connects it to the dermis (38). Different collagen types contribute to the integrated function of the BM. Collagen IV, the most abundant BM component, provides tensile strength (39). Collagen VII forms anchoring fibrils that extend from the BM into the dermis, stabilizing the dermal-epidermal connection (40). Collagen XVII is a key transmembrane protein in hemidesmosomes, linking the intracellular cytoskeleton to the BM and thereby reinforcing dermal-epidermal adhesion and overall skin structure (41). Our data revealed that UV irradiation reduced the expression of type IV, VII, and XVII collagen to 43%, 44%, and 41% of the control, respectively. Notably, the TC reversed these reductions, restoring their expression to 130%, 106%, and 114% of the baseline (Figures 3A, D–F), demonstrating its potent capacity to repair UV-damaged BM.

HA is another major ECM component that confers cushioning properties by forming hydrated networks with other ECM proteins, thereby helping to maintain structural integrity (42). The results showed that UV irradiation decreased HA levels to 29% of the control, while the TC restored HA levels to 114% of the baseline (Figures 3A, G), confirming its ability to recover the ECM's water-binding and water-retention capacity.

In summary, the TC effectively reversed the UV-induced loss of critical ECM components, including multiple collagen subtypes and HA. Importantly, it demonstrated superior repair efficacy over the individual ingredients and all dual combinations (Figures 3A–G), highlighting the protective advantage of the triple combination against ECM damage.

### 3.3 The TC regulates SASP secretion and activates the Nrf2/SOD2 axis against UV-induced damage

UV irradiation induces persistent oxidative stress, characterized by the excessive accumulation of ROS, which is a key driver of skin photoaging. To elucidate the underlying protective mechanisms of the TC, we analyzed the expression of antioxidant and inflammatory biomarkers.

**Figure 4:**
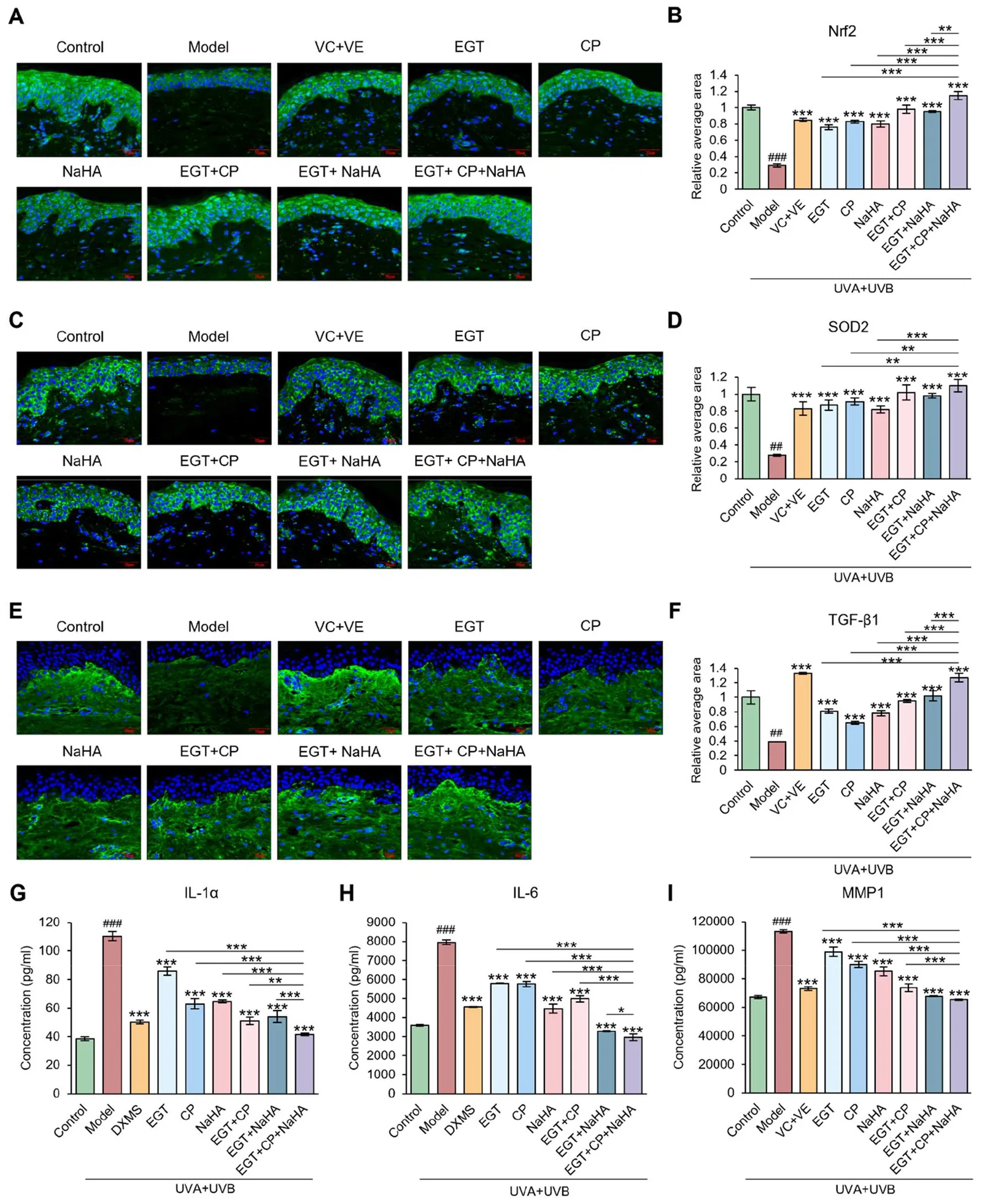
TC improve antioxidated ability and SASP secretion induced by UV irradiation. **(A, B)** Immunofluorescence and their quantitative results show the concentration of Nrf2, **(C, D)** SOD2, and **(E, F)** TGF-β1; **(G)** ELISA assay shows the concentration of IL-1α, **(H)** IL-6, and **(I)** MMP1. Samples in every groups *n* = 3. Statistical analysis was performed using Student's *t*-test. ## Indicates *p*-value <0.01, ### indicates *p*-value <0.001 compares with control groups; * indicates *p*-value <0.05, ** indicates *p*-value <0.01, *** indicates *p*-value <0.001 compares with model groups.

Our results showed that UV irradiation significantly downregulated the expression of nuclear factor erythroid 2-related factor 2 (Nrf2) and superoxide dismutase 2 (SOD2) in the model group, indicating a compromised cellular antioxidant defense system (Figures 4A–D). Conversely, this suppression was countered by all treatment groups, including the individual components (EGT, CP, NaHA), their dual combinations, and the triple combination, with the TC group showing the most potent effect (Figures 4A–D). These results demonstrate that all the treatments, particularly the triple combination, enhanced cellular antioxidant capacity and mitigated ROS-induced damage.

We further investigated whether the combination maintains normal ECM homeostasis by modulating the SASP. Quantitative analysis of key immune mediators revealed that TC treatment specifically restored the UV-downregulated TGF-β1 levels, while simultaneously inhibiting the UV-induced overexpression of IL-1α and IL-6 (Figures 4E–H). Additionally, a significant downregulation of MMP-1 was observed, indicating a reduction in collagen degradation (Figure 4I).

Collectively, these findings demonstrate that the TC exerts dual protective effects against UV-induced photoaging: it enhances antioxidant capacity by activating the Nrf2/SOD2 axis and maintains ECM integrity by regulating SASP-related inflammatory responses and collagen degradation.

### 3.4 The TC improves facial skin appearance and barrier function in human trial

Skin appearance and barrier function integrity are determined by various key parameters. In this clinical study, we systematically evaluated the repair efficacy of the orally administered TC by assessing skin ultrasound features, mechanical properties, hydration, and smoothness.

**Figure 5:**
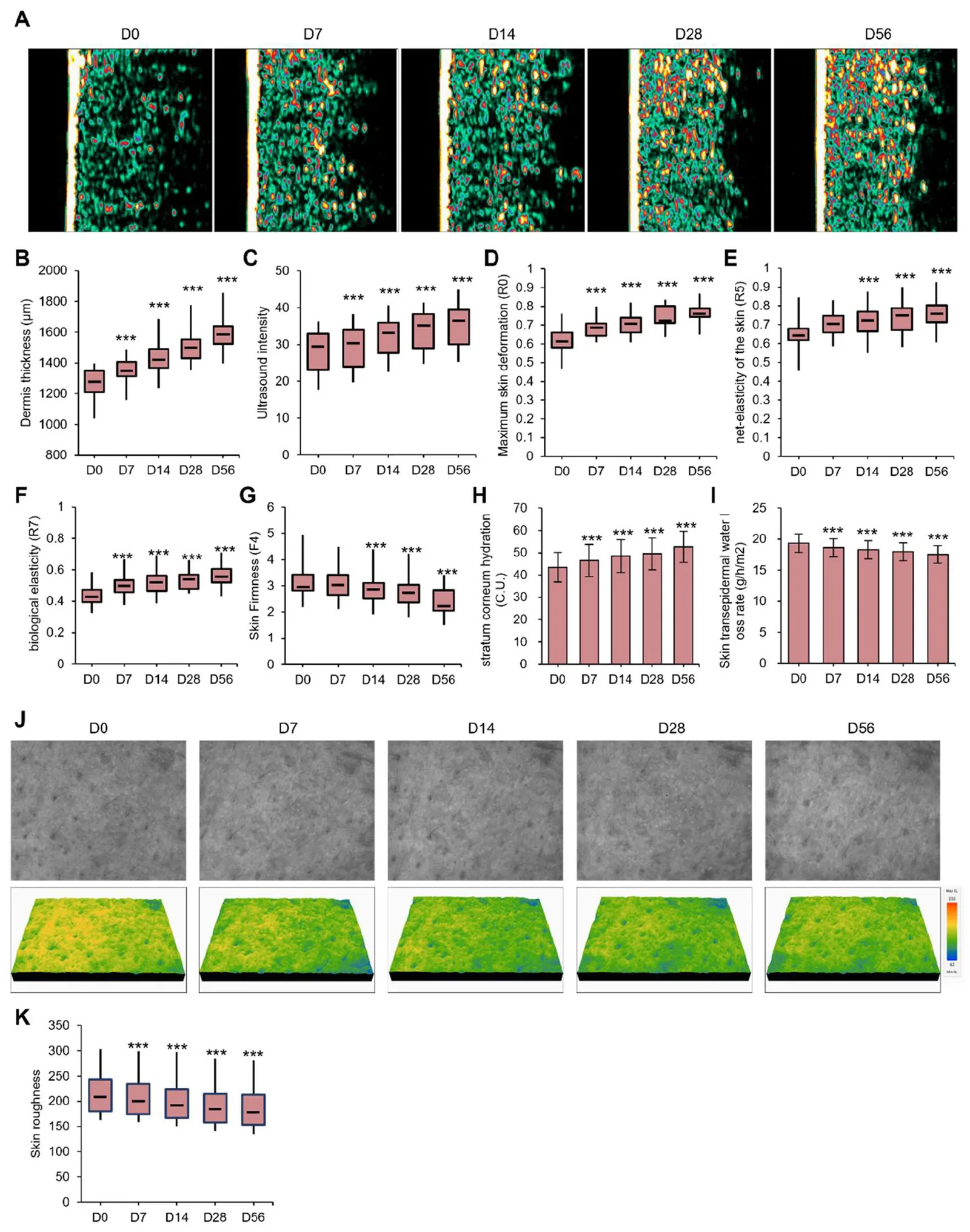
Overall condition of facial skin is improved after 0, 7, 14, 28, 56 days (D0, D7, D14, D28, and D56) of oral administration TC. Representative images **(A)**, skin thickness **(B)** and intensity score **(C)** produced from the ultrasound probe of DermaLab^®^ Combo; maximum skin deformation **(D)**, ration immediate retraction/immediate deformation **(E)**, ratio immediate retraction/maximum deformation **(F)** and the area value within the envelope curve formed during the periodic application and elimination of negative pressure **(G)** measured by Cutometer^®^ MPA580; **(H)** stratum corneum hydration measured by Corneometer^®^ CM825; **(I)** skin transepidermal water loss rate measured by Tewameter^®^ TMHex; representative images and 3D bump map **(J)** and skin smoothness score **(K)** produced from Visioscan^®^ VC20plus. *** Indicates *p*-value <0.001 compares with D0 group.

Skin thickness and collagen density were assessed using DermaLab^®^ Combo. The results showed an increase in both epidermal thickness and collagen density following treatment with the TC (Figures 5A–C). Mechanical properties assessed by the Cutometer^®^ MPA580 revealed notable improvements in skin elasticity and firmness, as reflected by changes in key elasticity-related parameters (Figures 5D–G). Furthermore, the combination treatment significantly increased stratum corneum hydration (SH) and reduced TEWL (Figures 5H, I), demonstrating its ability to enhance skin moisture retention and strengthen the skin barrier. As a comprehensive indicator of skin surface quality, skin roughness was also evaluated and a significant reduction was observed after treatment (Figures 5J, K).

Collectively, these findings demonstrate that the oral TC progressively improves facial skin appearance (including structural density, elasticity, and smoothness) and restores skin barrier integrity in a time-dependent manner, with efficacy becoming more pronounced as the intervention period extends.

### 3.5 The TC reduces facial wrinkles in human trial

To further comprehensively evaluate the anti-aging efficacy of the TC, a systematic assessment of multiple facial wrinkle types was conducted using standardized imaging and quantitative analysis tools.

**Figure 6:**
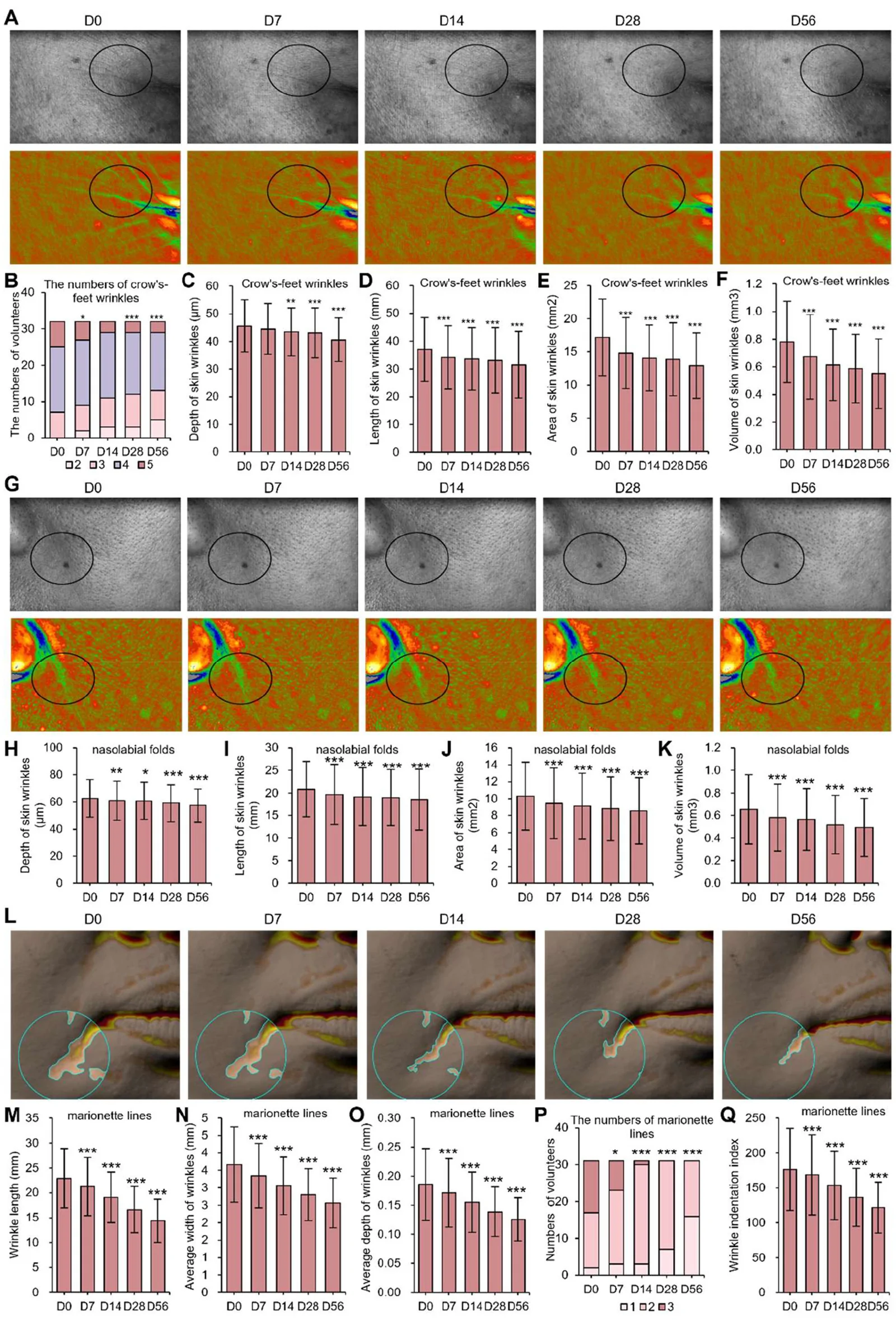
The TC oral administration after 0, 7, 14, 28, 56 days (D0, D7, D14, D28, and D56) shows the improvement in crow's feet wrinkles, nasolabial folds and marionette lines. Representative images **(A)**, number **(B)**, depth **(C)**, length **(D)**, area **(E)**, and volume **(F)** of crow's-feet wrinkles produced from the Primos CR; representative images **(G)**, depth **(H)**, length **(I)**, area **(J)**, and volume **(K)** of nasolabial produced from the Primos CR; representative images **(L)**, length **(M)**, average width **(N)**, average depth **(O)**, number **(P)**, and indentation index **(Q)** of marionette lines produced from the Antera 3D^®^. * Indicates *p*-value <0.05, ** Indicates *p*-value <0.01, *** indicates *p*-value <0.001 compares with D0 group.

Representative images and quantitative analysis showed that the combination treatment notably reduced the number, depth, length, area, and volume of crow's feet wrinkles by 12%, 11%, 15%, 25%, and 29%, respectively (Figures 6A–F). Furthermore, improvements in nasolabial folds and marionette lines were also observed, showing pronounced, time-dependent effects as measured by comprehensive indices (Figures 6G–Q). For nasolabial folds, the depth, length, area, and volume were reduced by 8%, 11%, 17%, and 24%, respectively (Figures 6H–K). Similarly, the combination treatment led to remarkable reductions in marionette lines: length (−37%), average width (−30%), average depth (−32%), number (−38%), and indentation index (Figures 6M–Q).

Collectively, these findings confirm that the TC exerts potent and comprehensive anti-wrinkle effects, effectively improving multiple types of facial wrinkles (crow's feet, nasolabial folds, marionette lines) that are hallmark manifestations of facial skin aging.

## 4 Discussion

Oral administration of EGT, CP, and NaHA has potential benefits. Oral EGT can sustainably increase its concentration in the blood for more than 4 weeks after discontinuation, with no adverse effects, and exhibits potential antioxidant capacity (43). Oral CP, especially low-molecular-weight CP, has been widely reported to benefit the skin and is believed to improve skin integrity, thereby combating skin aging (21, 44). Oral NaHA has also been reported to improve skin condition in various aspects, making it a good oral nutricosmetic ingredient (29). These findings suggest that the three components have the potential to form an oral combination for anti-aging purposes. This study aims to investigate the enhanced effect of the combination of these three components on skin anti-aging improvement.

The degenerative change in skin barrier function is the typical phenomenon of skin aging. The skin barrier function is crucial for maintaining skin integrity, helping the body prevent moisture and nutrient loss while resisting external insults. In UV irradiation models, *ex vivo* human skin typically exhibits epidermal thinning (45), whereas mouse models often show epidermal hyperplasia (46). Regardless of the model used, UV exposure is consistently accompanied by photodegradation of epidermal cells, decreased cell density, and extracellular damage (47). Similarly, skin aging is also associated with thinning of the epidermal layer (48, 49). These alterations ultimately contribute to impaired skin barrier function. The degeneration of the skin barrier is characterized by SH and increased TEWL. These are directly related to changes in stratum corneum lipid composition and abnormal keratinocyte proliferation and differentiation (48, 50).

HA is predominantly located in the dermal layer. In this layger, it is embedded within and surrounded by fibrous and matrix components. It interacts with matrix proteins and glycoproteins and can bind water up to 1,000 times its own volume, thereby maintaining dermal hydration (36, 50, 51). As a member of the aquaporin family, AQP3 mediates the transport of water and glycerol in skin cells and plays a key role in regulating cellular hydration (52). For instance, in both intrinsic aging and photoaging, the skin's ability to retain moisture is diminished, corresponding to a reduction in AQP3 mRNA and protein expression (53). In this study, we observed that the TC restored skin thickness and epithelial cell number, improved skin hydration and TEWL, and increased HA content and AQP3 expression. These results suggest that the TC reduces epidermal thinning caused by epidermal cell degeneration, helps maintain skin moisture, and thereby contributes to the recovery of skin barrier function.

The imbalance of the ECM in the dermis is a central driver of skin aging, as the ECM is essential for maintaining the structural integrity and physiological functions of skin (3, 54). Collagen accounts for more than 30% of the dry weight of the ECM (55). Although collagen types vary greatly in their abundance within the skin's ECM, all contribute crucially to skin stability (56). Collagen I and III, the first and second most abundant collagens in the body, constitute the majority of the dermal ECM. They determine skin rigidity, elasticity, and flexibility and are notably reduced in aged skin (57–59). As a major component of the basement membrane, the non-fibrillar collagen IV forms mesh-like networks within the ECM, functioning to support tissue architecture and enhance structural stability (60–62). Other collagens, including collagen VII and XVII, play vital roles in maintaining skin integrity. Collagen VII, the main component of anchoring fibrils, is synthesized by both keratinocytes and fibroblasts and secures the attachment of the epidermal basement membrane to the underlying dermal ECM (63, 64). Clinically, mutations in the collagen VII gene are strongly linked to epidermolysis bullosa, underscoring its importance for skin integrity (65, 66). Similarly, collagen XVII is a key structural element of hemidesmosomes, which anchor basal keratinocytes to the basement membrane (67). Clinical evidence further associates collagen XVII with epithelial adhesion (68–70), and its dysfunction can compromise skin integrity, leading to tissue loosening, multilayering, and potential rupture (67). In this study, we observed that the TC counteracted the reduction in collagen concentration and density induced by aging. It also enhanced skin elasticity and improved several types of wrinkles. These findings support the established understanding that age-related epidermal thinning significantly impairs skin function. As the main structural protein of the skin, the loss or fragmentation of collagen leads to decreased skin elasticity, firmness, and resilience, along with the appearance of wrinkles and fine lines (49). Therefore, restoring collagen within the ECM presents a promising strategy for addressing these clinically observable signs of aging. In our clinical study, although we selected objective indicators without self-reported evaluation, the lack of a placebo and insufficient blinding remain limitations of the pre-post self-controlled design, which weaken the validity of the conclusions to some extent. In the future, we will further address these limitations.

Aging is characterized by the accumulation of excessive ROS. A key consequence is the induction of the SASP, which promotes chronic inflammation and ECM degradation, thereby accelerating the aging process. Both the aging process and UV irradiation lead to a decreased ability to clear ROS, due in part to reduced nuclear translocation of the transcription factor Nrf2 (71, 72). Declining Nrf2 activity is recognized as a driver of multiple hallmarks of aging (73). Notably, Nrf2 inducers have been shown to prevent cellular senescence and reverse aging phenotypes in fibroblasts (72). Moreover, Nrf2 deficiency reduces the expression of COL1A1 and COL3A1 and inhibits the assembly of collagen fibers (74, 75). Functionally, Nrf2 binds to antioxidant response elements (AREs) and upregulates a suite of antioxidant genes, including superoxide dismutase (SOD), to mitigate ROS (76). SOD2, also known as manganese superoxide dismutase (MnSOD), constitutes the first line of defense against ROS. Its deficiency has been linked to reduced collagen content, ECM degradation, and decreased dermal thickness, underscoring its association with the skin aging phenotype (77, 78).

The immunosuppressive factor TGF-β1 and the pro-inflammatory factors IL-1α and IL-6 are also members of SASP, contributing to age-related decline in tissue function (79). TGF-β1 exhibits both anti-inflammatory and pro-inflammatory effects (80). Elevated TGF-β levels are involved in the chronicity of the skin wound healing, indicating a link between TGF-β mediated inflammation and skin repair processes (80). Meanwhile, increased IL-1α and IL-6 levels promote tissue inflammation, disrupt skin structure, and stimulate the overexpression of MMPs (50, 81). UV-induced IL-1α secretion further promotes IL-6 production, accelerating collagen enzymatic degradation by enhancing MMP1 transcription and translation, showing the connection between inflammatory response and ECM integrity (3, 82, 83).

In the present study, the TC reversed UV-induced reductions in skin antioxidant capacity, attenuated inflammatory responses, and protected the ECM from SASP-mediated degradation. This enhanced effect likely stems from the complementary mechanisms of the three components: EGT activates the Nrf2/SOD2 antioxidant axis to scavenge ROS, CP promotes collagen synthesis and inhibits MMP-mediated degradation, and NaHA enhances skin hydration and reinforces the barrier function. However, further investigation should employ more representative models for subsequent *in vivo* experiments in order to compensate for the limitation that *ex vivo* human skin models cannot reflect the effects of cutaneous microcirculation and systemic metabolism. Together, these findings demonstrate that the triple combination targets multiple interconnected pathways of skin aging—oxidative stress, inflammation, and ECM imbalance—offering a comprehensive and effective strategy for mitigating both photoaging and chronological aging.

## 5 Conclusion

In conclusion, this study identified a promising oral anti-aging combination of EGT, CP, and NaHA for facial skin. Through a comprehensive dual-validation strategy involving an *ex vivo* human skin model and a clinical trial in Chinese women, we systematically demonstrated that this triple combination exerts significant improved efficacy: it effectively prevents UV-induced collagen loss (including multiple key collagen subtypes and hyaluronic acid), enhances skin antioxidant capacity by activating the Nrf2/SOD2 axis, and mitigates chronic inflammation by regulating the SASP. These findings not only expand the current understanding of the individual and anti-aging mechanisms of EGT, CP, and NaHA, but also present a multidimensional and translatable strategy for addressing both intrinsic chronological aging and extrinsic photoaging. Future research should focus on elucidating the precise molecular crosstalk underlying the additional benefit of the three components, expanding the sample size and extending the follow-up period in clinical trials, and optimizing the formulation ratio to further improve efficacy, thereby providing stronger scientific support for the development of next-generation oral nutricosmetics for skin anti-aging.

## Data Availability

The raw data supporting the conclusions of this article will be made available by the authors, without undue reservation.
