# Supplementary material for: Ergothioneine, collagen peptides, and sodium hyaluronate ameliorate facial skin aging via modulating oxidative stress, inflammation, and extracellular matrix homeostasis
Source: Front Nutr. 2026 Aug 21;13:1888269. doi: 10.3389/fnut.2026.1888269 (PMC13541516; doi:10.3389/fnut.2026.1888269)
Supplement: Supplementary file 1 [file Image_1.pdf]

Supplementary Material

1 Supplementary Data

1.1 Supplementary Figures

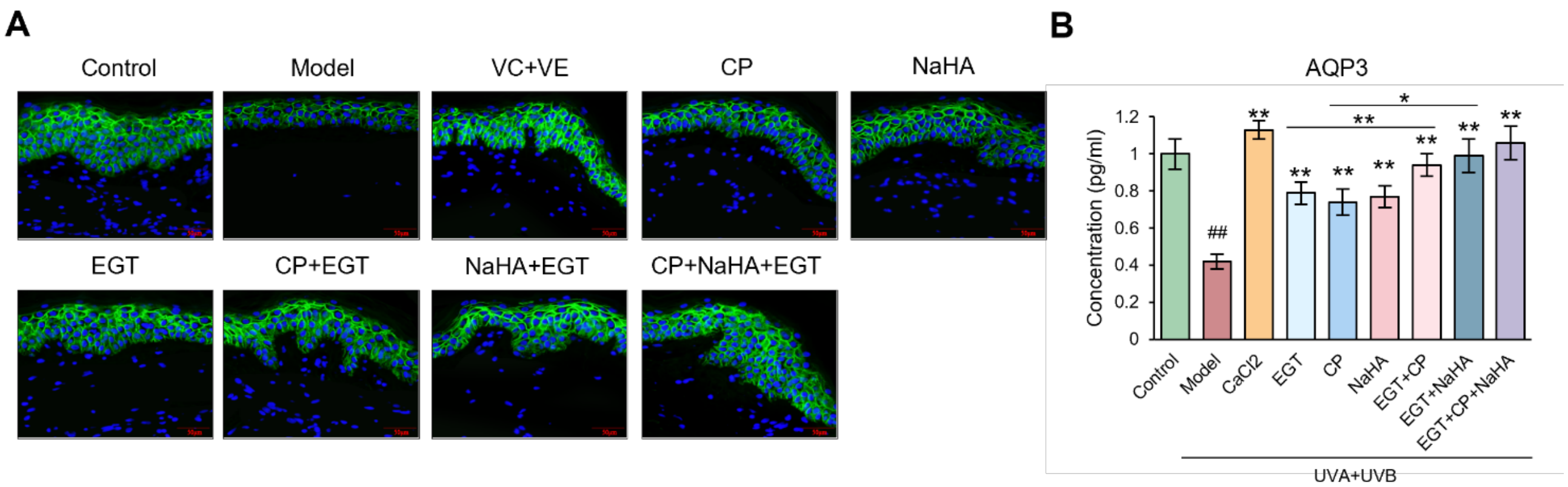

Supplementary Figure 1. EGT+CP+NaHA combination improve water-retention capacity. (A,B) Immunofluorescence and their quantitative results show the concentration of AQP3. ## indicates p-value < 0.01 compares with control groups; * indicates p-value < 0.05, ** indicates p-value < 0.01 compares with model groups.
